# Supplementary material for: Overexpression of Canonical Prefoldin Associates with the Risk of Mortality and Metastasis in Non-Small Cell Lung Cancer
Source: Cancers (Basel). 2020 Apr 24;12(4):1052. doi: 10.3390/cancers12041052 (PMC7225921; doi:10.3390/cancers12041052)
Supplement: Supplementary file 1 [file cancers-12-01052-s001.pdf]

cancers

MDPI

Supplementary Materials: Overexpression of Canonical Prefoldin Associates with the Risk of Mortality and Metastasis in Non-Small Cell Lung Cancer

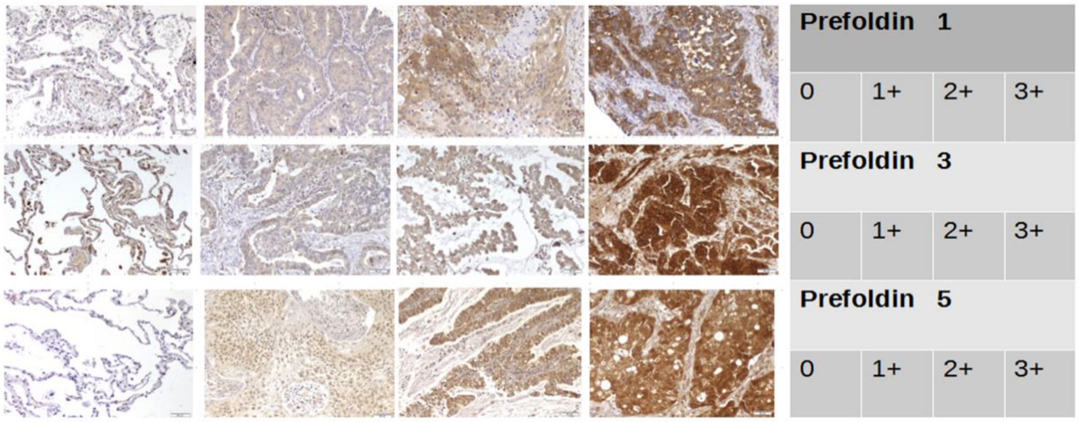

Figure S1. Prefoldin expression was semi-quantitatively scored by two independent pathologists on a range of 0 to 3+. Scale bar: 50 μm.

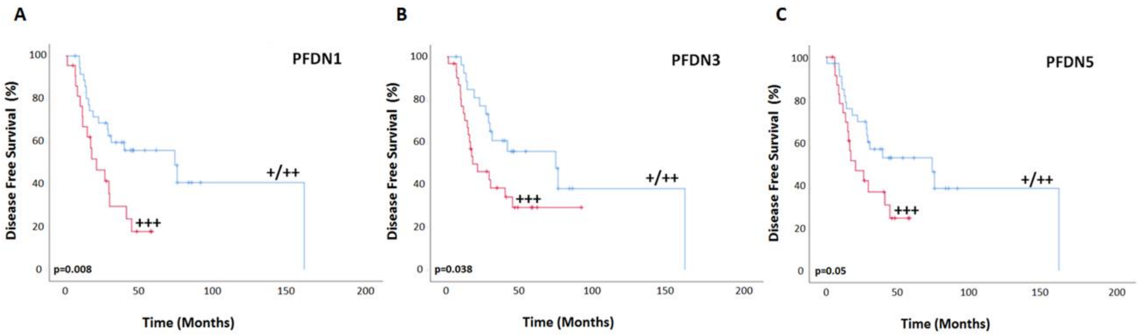

Figure S2. Kaplan–Meier curves of all 58 patients for disease-free survival (DFS) according to PFDN expression. Patients with PFDN1 (**A**), 3 (**B**) and 5 (**C**) overexpression had lower DFS.

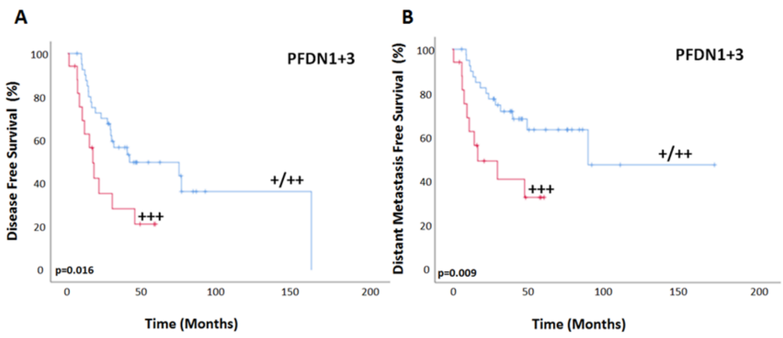

Figure S3. Kaplan–Meier curves of all 58 patients for (**A**) disease-free survival (DFS) and (**B**) distant metastasis-free survival (DMFS) when combining all overexpressed PFDNs. Patients with PFDN1 and 3 overexpression had lower (**A**) DFS and (**B**) DMFS.

Table S1. Univariate analyses of the associations between patient characteristics and prefoldin (PFDN) protein expression levels and the outcome.

Parameter	Overall Survival			Disease-Free Survival			Local Recurrence			Distant Metastases		
	HR	95% CI	*p* value	HR	95% CI	*p* value	HR	95% CI	*p* value	HR	95% CI	***p*-value**
Gender												
Male (Ref)	1			1			1			1		
Female	0.47	0.14–1.56	0.221	0.84	0.29–2.40	0.751	0.50	0.11–2.18	0.360	0.98	0.29–3.33	0.985
Age. years												
≤67 (Ref)	1			1			1			1		
>67	0.83	0.43–1.59	0.584	0.80	0.59–1.41	0.695	0.71	0.30–1.70	0.451	0.70	0.31–1.60	0.410
COPD												
No (Ref)	1			1			1			1		
Yes	0.99	0.52–1.89	0.984	0.58	0.28–1.21	0.149	1.13	0.47–2.72	0.773	0.49	0.20–1.20	0.122
Cardiovascular disease												
No (Ref)	1			1			1			1		
Yes	1.89	0.92–3.87	0.079	1.37	0.62–3.062	0.430	1.31	0.47–3.59	0.600	2.28	0.96–5.40	0.059
Smoking status												
Former/Never (Ref)	1			1			1			1		
Current	1.36	0.72–2.58	0.340	0.70	0.35–1.42	0.336	0.69	0.28–1.67	0.419	1.04	0.46–2.33	0.917
Alcohol												
No (Ref)	1			1			1			1		
Yes	1.14	0.59–2.21	0.682	0.97	0.48–1.94	0.934	0.70	0.29–1.70	0.437	1.34	0.59–3.05	0.476
KPS												
≥80 (Ref)	1			1			1			1		
<80	1.63	0.80–3.322	0.179	0.86	0.35–2.10	0.747	0.88	0.29–2.65	0.820	0.81	0.27–2.40	0.713
Weight												
<78 (Ref)	1			1			1			1		
≥78	0.60	0.31–1.16	0.133	0.46	0.22–0.94	0.034	0.63	0.26–1.52	0.307	0.27	0.10–0.69	0.006
Body Surface												
<188 (Ref)	1			1			1			1		
≥188	0.41	0.21–0.81	0.010	0.46	0.22–0.93	0.033	0.85	0.35–2.04	0.726	0.22	0.08–0.56	0.002
Tumor Histology												
Adeno (Ref)	1			1			1			1		
Squamous cell	0.92	0.48–1.78	0.821	0.76	0.38–1.52	0.445	1.60	0.64–4.02	0.314	0.49	0.22–1.12	0.092

Clinical stage												
I - II (Ref)	1			1			1			1		
III – IV	2.04	0.94–4.42	0.069	2.12	0.94–4.74	0.067	1.28	0.51–3.23	0.596	3.94	1.17–13.29	0.027
Surgery												
No (Ref)	1			1			1			1		
Yes	0.42	0.17–1.04	0.061	0.51	0.17–1.46	0.210	0.71	0.16–3.09	0.651	0.34	0.11–1.00	0.051
Chemotherapy												
No (Ref)	1			1			1			1		
Yes	1.55	0.75–3.18	0.231	1.75	0.78–3.89	0.170	1.30	0.50–3.37	0.590	4.59	1.34–15.75	0.015
Concomitant chemotherapy												
No (Ref)	1			1			1			1		
Yes	3.31	1.38–7.93	0.007	2.51	0.94–6.68	0.066	2.16	0.61–7.70	0.231	3.88	1.38–10.91	0.010
Radiotherapy												
No (Ref)	1			1			1			1		
Yes	1.84	0.92–3.66	0.082	1.59	0.79–3.2	0.189	1.21	0.49–2.96	0.667	3.39	1.44–7.96	0.015
PFDN1*												
0/+/++ (Ref)	1			1			1			1		
+++	2.86	1.47–5.56	0.002	2.53	1.24–5.15	0.010	1.68	0.67–4.23	0.266	2.94	1.28–6.73	0.011
PFDN3*												
0/+/++ (Ref)	1			1			1			1		
+++	2.30	1.17–4.50	0.015	2.09	1.02–4.27	0.042	1.50	0.62–3.63	0.362	2.50	1.06–5.88	0.036
PFDN5*												
0/+/++ (Ref)	1			1			1			1		
+++	2.94	1.50–5.76	0.002	2.00	0.98–4.06	0.055	1.74	0.70–4.32	0.227	1.94	0.85–4.42	0.112

Abbreviations: HR. hazard ratio; COPD. Chronic obstructive pulmonary disease; KPS. Karnofsky performance status; PFDN. Prefoldin. * (+), weak expression; 2 (++), strong expression, and 3 (+++), very strong expression

Table S2. Multivariate analyses of the associations between patient characteristics and PFDN expression and outcome.

Parameter	Overall Survival			Disease Free Survival			Distant Metastases		
	**HR**	**95% CI**	**p value**	**HR**	**95% CI**	p value	HR	95% CI	p value
Body Surface									
<188 (Ref)							1		
≥188							0.27	0.10-0.72	0.009
Concomitant Chemotherapy									
No (Ref)	1								
Yes	3.76	1.55-9.11	0.003						
Radiotherapy									
No (Ref)							1		
Yes							3.44	1.42-8.34	0.006
PFDN1									
0/+/++ (Ref)				1			1		
+++				2.53	1.24-5.15	0.010	2.32	0.98-5.45	0.053
PFDN5									
0/+/++ (Ref)	1								
+++	3.13	1.59-6.15	0.001						

Abbreviations: HR. hazard ratio; PFDN. prefoldin. * (+), weak expression; 2 (++), strong expression, and 3 (+++), very strong expression
